# Effect of Active Coatings Containing *Lippa citriodora* Kunth. Essential Oil on Bacterial Diversity and Myofibrillar Proteins Degradation in Refrigerated Large Yellow Croaker

**DOI:** 10.3390/polym13111787

**Published:** 2021-05-28

**Authors:** Bo Li, Xuesong Wang, Xin Gao, Jun Mei, Jing Xie

**Affiliations:** 1 College of Food Science and Technology, Shanghai Ocean University, Shanghai 201306, China; d190300060@st.shou.edu.cn (B.L.); m180310728@st.shou.edu.cn (X.W.); d180300061@st.shou.edu.cn (X.G.); 2 National Experimental Teaching Demonstration Center for Food Science and Engineering, Shanghai Ocean University, Shanghai 201306, China; 3 Shanghai Engineering Research Center of Aquatic Product Processing and Preservation, Shanghai 201306, China; 4 Shanghai Professional Technology Service Platform on Cold Chain Equipment Performance and Energy Saving Evaluation, Shanghai 201306, China

**Keywords:** active coating, large yellow croaker, lemon verbena essential oil, bacterial diversity, myofibrillar proteins structure

## Abstract

The research evaluated the effects of locust bean gum (LBG) and sodium alginate (SA) active coatings containing 0.15, 0.30 or 0.60% lemon verbena (*Lippa citriodora* Kunth.) essential oil (LVEO) on the bacterial diversity and myofibrillar proteins (MPs) of large yellow croaker during refrigerated storage at 4 °C for 18 days. Variability in the dominant bacterial community in different samples on the 0, 9th and 18th day was observed. *Pseudomonas* and *Shewanella* were the two major genera identified during refrigerated storage. At the beginning, the richness of *Pseudomonas* was about 37.31% and increased for control (CK) samples during refrigerated storage, however, the LVEO-treated samples increased sharply from day 0 to the 9th day and then decreased. LBG-SA coatings containing LVEO treatments significantly delayed MPs oxidation by retarding the formation of free carbonyl compounds and maintaining higher sulfhydryl content, higher Ca^2+^-ATPase activity, better organized secondary (higher contents of α-helix and β-sheet) and tertiary structures during refrigerated storage. The transmission electron microscope (TEM) images showed that the integrity of the sarcomere was damaged; the boundaries of the H-, A-, and I-bands, Z-disk, and M-line were fuzzy in the CK samples at the end of storage. However, the LVEO-treated samples were still regular in appearance with distinct dark A-bands, light I-bands, and Z-disk. In brief, LBG-SA active coatings containing LVEO treatments suggested a feasible method for protecting the MPs of large yellow croaker during refrigerated storage.

## 1. Introduction

The large yellow croaker (*Pseudosciaena crocea*) is known as one of the most commercially and economically valuable marine fish in the China [1]. It is a kind of delicious fish with high nutritional value. However, large yellow croaker is highly susceptible to decay under the action of microorganisms and related enzymes, resulting in protein degradation, lipid oxidation, and undesirable compounds, which lead to a relatively short shelf life [2]. Therefore, it is important to maintain the good quality of fresh large yellow croaker and delay its deterioration during storage. Many bio-preservatives, such as bacteriocin [3], lysozyme [4], epsilon-polylysine and rosemary extract [5], apple polyphenols [6], bayberry leaf extract [7], and *Allium sativum* essence oil [8] have been applied in large yellow croaker preservation.

Active packaging refers to the packaging technology in which active agents are added into the packaging system to reduce or inhibit the growth of microorganisms in food [9]. Locust bean gum (LBG), extracted from the seeds of *Ceratonia siliqua* carob tree, is a potential coating component due to its excellent active coating-forming properties and its ability to form strong gels at relatively low concentrations [10]. LBG is composed of a β-(1-4)-d-mannopyranosyl backbone with α-d-galactopyranosyl replaced by C_6_ of mannose and therefore they are referred to as the galactomanan. The ratio of mannose to galactose in LBG is about 3.5:1 [11]. LBG can form viscous solutions at relatively low concentrations and produce dense coatings with excellent mechanical and vapor barrier properties, which has been extensively researched for coating applications [12,13]. Sodium alginate (SA) is an algal carbohydrate macromolecule with potential film-forming properties. It is an anionic copolymer of alginic acid including (1–4)-β-d-mannuronate and α-l-guluronate residues covalently linked together in different sequences [14]. SA is non-toxic, renewable, and biodegradable and can be used in the production of active coating as a polymer matrix for the substance to be released [15]. The coatings obtained from SA are uniform and transparent, however, SA-based coatings exhibit poor moisture barrier properties due to the high hydrophilicity [16]. LBG is non-ionic and its aqueous solubility is not affected by pH or ionic strength of the liquid medium [17]. SA could be used as an ion source (anionic) to promote the mucoadhesive property of non-ionic LBG [18].

The active coating contains antimicrobial agents that inhibit the spoilage and oxidation of food more effectively compared with the method of adding the active substance directly into the food formulation [19]. Due to the growing consumer demand for natural products, essential oils (EOs) are gaining in popularity [20,21] for the antimicrobial and antioxidant properties to extend the shelf life of foods. Loke et al. [22] reported collagen active packaging containing 6% cinnamaldehyde, which inhibited the growth of microorganisms and delayed the production of total volatile basic nitrogen (TVB-N), and thiobarbituric acid reactive substances (TBARS) during cold storage at 4 °C to extend the shelf life of tilapia fillets to 3 days. Homayonpour et al. [23] showed that nanochitosan degradable coating with nano-encapsulated *Cumino cyminum* L. essential oil effectively inhibited the growth of microorganisms and the chemical spoilage reflected at lower pH, peroxide value, and TBARS for sardine fillets during refrigerated storage. Specifically, lemon verbena (*Lippa citriodora* Kunth.) essential oil (LVEO) has received much attention because of its antimicrobial and antioxidant properties [24,25]. Lemon verbena is an important aromatic plant, mainly due to the lemon-like aroma exuding from its leaves, and is usually used to prepare the herbal teas [26]. Previous research showed its beneficial effects, including antimicrobial, cardioprotective, neuroprotective, anti-inflammatory, anticonvulsant, and antigenotoxic [27,28]. The LVEO exhibits antimicrobial activity and could be applied to food preservation. Rezaeifar et al. [29] reported that the chitosan coating incorporated with LVEO significantly reduced microbial growth and had an agreeable effect on sensory characteristics during refrigerated storage at 4 °C, which can be used as an alternative to chemical preservatives in fish storage. Hosseini et al. [30] found that sodium alginate active coating containing 0.5% LVEO in modified atmosphere packaging (65% CO_2_, 30% N_2_, and 5% O_2_) is the most effective combination in lowering both gram-positive and gram-negative tested bacteria and plays an important role in increasing the shelf life of chicken breast. Neral, geraniol, 1,8-cineole, and limonene are the most important volatile compounds of LVEO and are natural monoterpens [31]. Neral is the main active compound in LVEO and has high antioxidant and fungicidal properties. The antimicrobial activity of neral against several food pathogens has been well documented in in-vitro trials [32].

In this research, the effects of LBG and SA active coatings containing LVEO on the bacterial diversity and inhibiting myofibrillar proteins (MPs) oxidation and degradation of large yellow croaker samples during refrigerated storage at 4 °C were explored.

## 2. Materials and Methods

### 2.1. Preparation of LBG-SA Active Coating Solutions Containing LVEO Emulsions

The LVEO/lecithin emulsions were prepared according Liu et al. [33] with some minor modifications. An amount of 1.5 g of lecithin and different concentrations of LVEO additions (0.15%, 0.30%, and 0.60%, *v*/*v*, respectively) were stirred evenly at 40 °C. Then the deionized water was added and kept stirring for 6 h to obtain 100 mL of LVEO/lecithin emulsions. LBG (0.5% *w*/*v*, from *Ceratonia siliqua* seeds, >75% galactomannan content), SA (1.5% *w*/*v*, Mann/Gulu = 2:1, Mw 2.1 × 10^6^ g/mol, viscosity of 200 ± 20 mPa·s) and glycerol (0.6% *w*/*v*) were added to the prepared LVEO/lecithin emulsions (1 L) and stirred for 2 h to be dissolved. Then, the mixture was ultrasonically homogenized to generate homogeneous LBG-SA active coating solutions containing LVEO emulsions and degassed under vacuum.

### 2.2. Preparation of Large Yellow Croaker Samples

**Figure 1:**
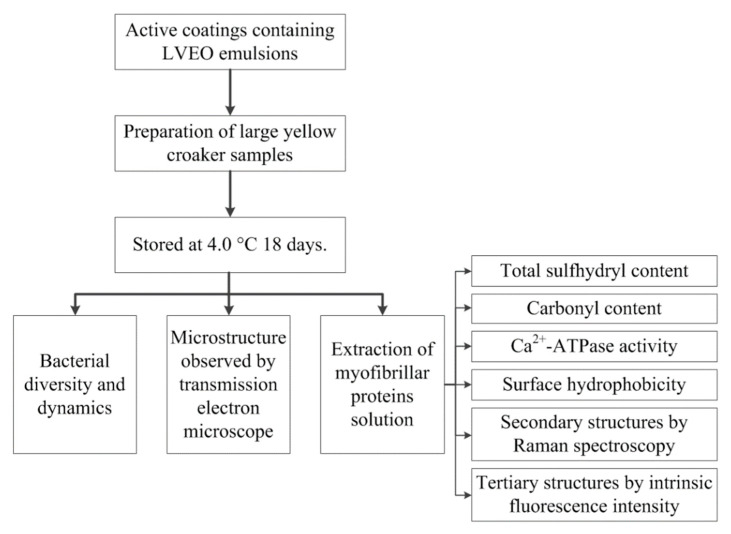
A schematic representation of preparation procedures.

Fresh large yellow croaker samples (700 ± 25 g) were randomly divided into four batches for (1) CK (large yellow croaker samples were packaged with LBG-SA active solution without LVEO); (2) LYC-0.15%LVEO (large yellow croaker samples were packaged with LBG-SA active solution containing 0.15% LVEO emulsion); (3) LYC-0.30%LVEO (large yellow croaker samples were packaged with LBG-SA active solution containing 0.30% LVEO emulsion); (4) LYC-0.60%LVEO (large yellow croaker samples were packaged with LBG-SA active solution containing 0.60% LVEO emulsion). The large yellow croaker samples with different treatments were packaged with the fresh-prepared active coatings solutions and then stored at 4.0 °C for subsequent testing every 3 days (Figure 1).

### 2.3. Analysis of Bacterial Diversity and Dynamics

Total genome DNA from large yellow croaker samples was extracted using CATB/SDS method developed by Biotree (Shanghai, China). 16S rRNA/18S rRNA/ITS genes of different regions (16S V4/16S V3/16S V3-V4/16S V4-V5, 18S V4/18S V9, ITS1/ITS2, Arc V4) were amplified used specific primer (e.g., 16S V4: 515F-806R, 18S V4: 528F-706R, 18S V9: 1380F-1510R, et al.) with the barcode. All PCR reactions were performed with 15 μL of Phusion^®^ High-Fidelity PCR Master Mix (New England Biolabs, MA, USA). The sequencing libraries were generated using TruSeq^®^ DNA PCR-Free Sample Preparation Kit (Illumina, CA, USA) at the manufacturer’s recommendations. The library quality was evaluated on Qubit@2.0 Fluorimeter (Thermo Scientific, MA, USA) and Agilent Bioanalyzer 2100 system. Finally, the library was sequenced on Illumina NovaSeq platform to generate 250 bp paired-end reads.

### 2.4. Extraction of MPs Solution

MPs solution was extracted by means of Yang et al. [34] with some modifications. Two grams of large yellow croaker flesh was homogenized with 20 mL pre-cooling Tris-buffer A (20 mM Tris-maleate, pH 7.0, 0.05 M KCl) and then the mixture was centrifuged at 16,560× *g* for 20 min at 4 °C. The supernatant was discarded and 20 mL pre-cooling Tris-buffer A was added again to repeat the aforementioned steps. The supernatant was discarded and the precipitate was homogeneously mixed with 20 mL pre-cooling Tris-buffer B (20 mM Tris-maleate, pH 7.0, 0.6 M KCl). Then the mixture was centrifuged at 16,560× *g* for 20 min at 4 °C and the supernatant was collected as MP solution.

#### 2.4.1. Determination of Total Sulfhydryl Content

One gram of large yellow croaker flesh was homogenized with 10 mL 8 M urea and 0.6 M NaCl solutions, and then the mixture was centrifuged at 2500× *g* for 10 min at 4 °C. Supernatant in an amount of 0.5 mL was mixed with 4.5 mL buffer C (pH 8.0, 0.2 M Tris-HCl, 8 M urea, 3 mM EDTA, 1% SDS), and 0.625 mL buffer D (pH 8.0, 10 mM Tris-HCl, 10 mM DTNB) was subsequently added. The mixture was incubated at 40 °C for 20 min and the absorbance was then determined at 412 nm. The content of sulfhydryl group was expressed as μmol/g protein.

#### 2.4.2. Determination of Free Carbonyl Compounds Contents

2, 4-dinitrophenyl hydrazine (DNPH) derivatization method was used to determine the free carbonyl compounds content with the method of Sun et al. [35]. Ten millimeters of DNPH was mixed with 1 mL MP solution at 25 °C for 1 h. The mixture was precipitated with 1 mL 20 % trichloroacetic acid (TCA) solution and centrifuged at 2500× *g* for 15 min at 4 °C. The supernatant was discarded and the precipitate was mixed with 1 mL ethanol: ethyl acetate (1:1, *v*/*v*) containing 10 mM HCl. Then the resulting pellet was incubated after dissolving in 6 M guanidine hydrochloride at 37 °C for 16 min. The absorbance was then measured at 370 nm and the free carbonyl compounds content was expressed as μmol/g protein.

#### 2.4.3. Determination of Ca^2+^-ATPase Activity

The activity of Ca^2+^-ATPase was measured with the method of Wang et al. [36]. An amount of 0.2 mL of Tris-maleate (0.5 M, pH 7.0), 0.2 mL CaCl_2_ (0.1 M), and 3 mL deionized water were added to the 0.4 mL MP solution (4 mg/mL). The mixture was activated by 0.2 mL 20 mM ATP. The system was incubated in 25 °C water bath for 3 min and stopped by 2 mL 15% TCA solution. Then the inorganic phosphate, which was gained by the centrifugation at 9000× *g* for 2 min, was quantified by the mixed solution of 3% ammonium molybdate, 3 M H_2_SO_4_, and 20% ascorbic acid in the same volumes. The activity of Ca^2+^-ATPase was expressed as μmol Pi/mg/min.

#### 2.4.4. Determination of Surface Hydrophobicity

The surface hydrophobicity of MPs was determined with the method of Zhang et al. [37]. Five gradients of MP solutions (0, 0.20, 0.40, 0.60, and 1.00 mg/mL) were prepared and 10 mL of each diluted MP solution was mixed with 25 μL ANS solution (8 mmol/L ANS, pH = 7.0). The mixture was reacted in 25 °C water bath for 10 min in the dark and the fluorescence intensity was measured using a microplate reader (MK3, Thermo) with the emission and excitation wavelengths of 485 and 374 nm, respectively. The slope of the linear equation between fluorescence intensity and corresponding MP concentration was described as the surface hydrophobicity (S_0_) of each MP solution.

#### 2.4.5. Raman Spectroscopy

A high-resolution Raman spectrometer (Labram HR Evolation, Longjumeau, France) was used to measure Raman spectra according to Cao et al. [38]. Samples with or without isotope H/D exchange were prepared. A sample of approximately 1 g MPs was placed on a glass slide and under the Raman microscope. The acquisition parameters were as follows: raster of 600 g/mm, resolution ratio of 2 cm^−1^, slit width of 200 μm, and an integral time of 60 s. The spectrum of 40–4000 cm^−1^ was obtained at the ratio of cm^−1^/min^−1^ in the Labspec 6.0 software (Horiba Scientific, Lille, France).

#### 2.4.6. Determination of Intrinsic Fluorescence Intensity (IFI)

The IFI of MPs samples were detected according to Tan et al. [39] and scanned with a fluorescence spectrophotometer (F-7100, Hitachi, Tokyo, Japan) in emission scanning mode. The experimental parameters were as follows: emission wavelength of 310–400 nm, excitation wavelength of 295 nm, slit width of 5 nm, and scan speed of 1200 nm/min. The maximum fluorescence wavelength (λ_max_) was recorded.

### 2.5. Microstructure Observed by Transmission Electron Microscope (TEM)

The TEM observation of large yellow croaker samples was performed with the method of Yang et al. [34]. The samples were cut into small squares (0.1 cm × 0.1 cm × 0.1 cm) and soaked in 2.5% glutaraldehyde. The samples were washed with 0.1 M PBS (pH 7.0) for three times and then dehydrated with gradient ethanol solutions (30, 50, 70, 80, 90, 95, and 100%, *v*/*v*). Next, they were soaked in epoxy resin and acetone (1:1, *v*/*v*) for 24 h, and then immersed in 100% epoxy resin overnight. After embedding in epoxy resin at 70 °C for 24 h, the samples were sliced into 70 nm and then stained with uranium acetate and lead citrate for 10 min. Afterwards, they were observed using a TEM (Hitachi HT 7700, Tokyo, Japan).

### 2.6. Statistical Analysis

The multiple comparisons were performed by one-way analysis of variance (ANOVA) using SPSS 22.0, and the results were expressed as means ± standard deviation.

## 3. Results and Discussions

### 3.1. Microbiome Analysis

#### 3.1.1. Community Abundance and Diversity

**Figure 2:**
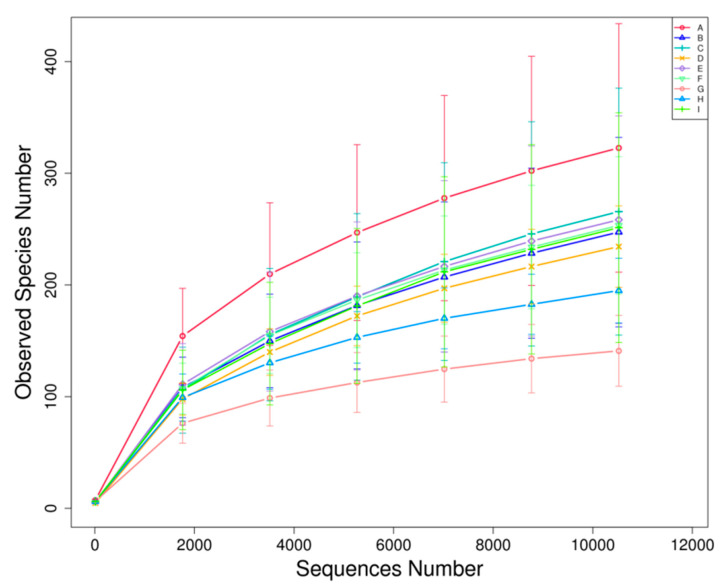
Rarefaction curves of 16S ribosomal RNA genes sequencing of bacterial V4 region of the large yellow croaker samples during refrigerated storage at 4 °C. A: fresh; B: CK on the 9th day; C: LYC-0.15%LVEO on the 9th day; D: LYC-0.30%LVEO on the 9th day; E: LYC-0.60%LVEO on the 9th day; F: CK on the 18th day; G: LYC-0.15%LVEO on the 18th day; H: LYC-0.30%LVEO on the 18th day; I: LYC-0.60%LVEO on the 18th day.

The calculated Good’s coverage values for the bacterial community abundance ranged from 0.989 to 0.993, indicating that the sample had a high coverage rate (Table 1). The sequencing results also indicated the bacterial diversity in large yellow croaker samples during refrigerated storage. The dilution curve of gene sequence V4 region (Figure 2) showed the sampling depth, and the curves under all storage treatments had gentle trends, demonstrating that the sequencing results could truly reflect the bacterial distribution in the large yellow croaker samples during refrigerated storage. Moreover, the number of bacteria species in the samples refrigerated and stored at 4 °C reached the maximum on the 9th day and then significantly decreased. The bacteria species number went down most sharply for the LYC-0.15%LVEO samples during refrigerated storage, however, the CK samples increased from the 9th day to the 18th day. This variation trend of the number of bacteria species based on the dilution curves with storage time was consistent with the results of biodiversity analysis (Table 1) in terms of OTUs, Chao (richness), ACE (uniformity), and Shannon (diversity index). Among them, the OTUs in LYC-0.15%LVEO samples on the 18th day were found to be the lowest. However, the fresh samples had the highest OTUs, Chao, and ACE indexes, which were 322, 425.722, and 459.919, respectively. These results suggested that the fresh samples could maintain the diversity of bacteria, while refrigerated storage might be more liable to form the dominant bacteria, resulting in a decrease in the diversity [40], especially for the LYC-0.15%LVEO samples.

#### 3.1.2. Relative Abundance of the Major Phyla and Genera

**Figure 3:**
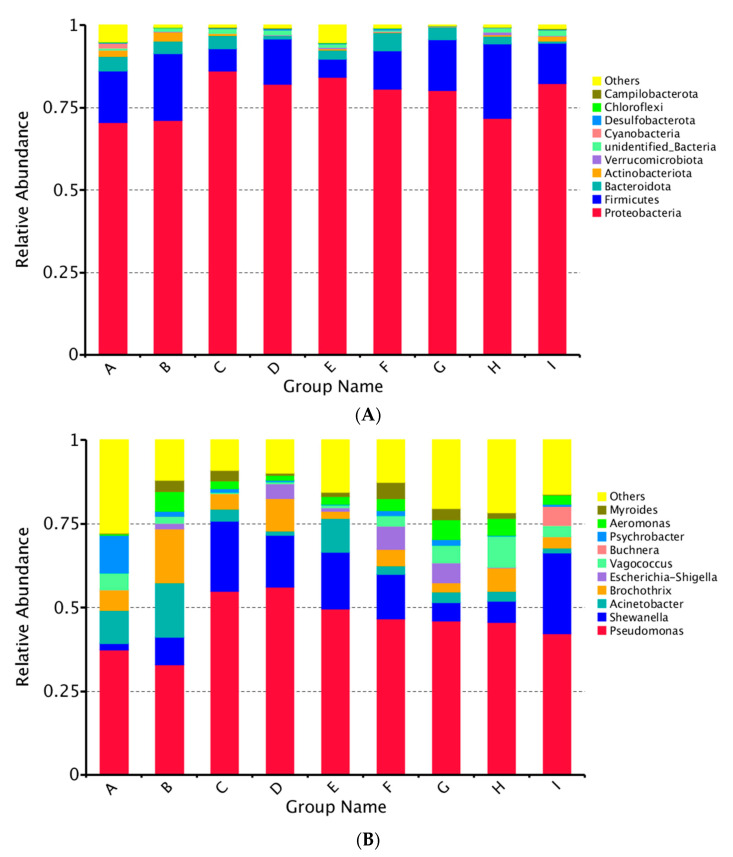
Microbial community structure variation in different stages. The relative abundance of bacteria at the (**A**) phylum and (**B**) genus level was shown. Below, the top 10 abundances at the phylum and genus levels were merged into others. Each bar represents the relative abundance of each sample. Each color represents a particular phylum or genus. A: fresh; B: CK on the 9th day; C: LYC-0.15%LVEO on the 9th day; D: LYC-0.30%LVEO on the 9th day; E: LYC-0.60%LVEO on the 9th day; F: CK on the 18th day; G: LYC-0.15%LVEO on the 18th day; H: LYC-0.30%LVEO on the 18th day; I: LYC-0.60%LVEO on the 18th day.

In order to evaluate the change of bacterial community structure, 16S rRNA was also used to analyze the abundance of individual bacterial species (phyla and genus) during storage. Figure 3 showed the top 10 most abundant species. Proteobacteria and Firmicutes were the most abundant bacterial phyla with a total relative abundance of more than 80%, followed by Bacteroidota and Actinobacteria (Figure 3A). On day 0, the relative abundance of Proteobacteria reached 70%, which was the key microbial phylum in large yellow croaker samples. When stored at 4 °C, the richness of Proteobacteria increased first and then decreased during the whole storage. The Proteobacteria reached the maximum value of 86.23% on the 9th day for LYC-0.15%LVEO and then dropped to 80.15% at the end. Meanwhile, the richness of Firmicutes increased from day 0 to the 9th day for CK samples and had an opposite trend to that of LVEO-treated samples during storage. The Proteobacteria for LYC-0.30%LVEO samples decreased from 15.62% on day 0 to 13.83% on the 9th day and increased to 22.70% on the 18th day.

At the genus level, *Pseudomonas* and *Shewanella* were the two major genera identified (Figure 3B). *Pseudomonas* was dominant over the storage period with the initial abundance of 37.31%. The richness increased for CK samples during refrigerated storage; however, the LVEO-treated samples increased sharply from day 0 to the 9th day and then decreased. For LYC-0.30%LVEO samples, the richness increased to 56.20% on the 9th day and decreased to 45.56% at the end of storage. *Shewanella* was another dominant microorganism with an initial abundance about 0.02%. The richness increased for CK and LYC-0.60%LVEO samples during refrigerated storage. The LYC-0.15%LVEO and LYC-0.30%LVEO samples increased from day 0 to the 9th day and then decreased. For LYC-0.60%LVEO samples, the richness increased to 24.07% at the end of storage. *Pseudomonas* and *Shewanella* are gram-negative psychrotrophic bacteria decaying fish in refrigeration and are considered to be the main spoilage bacteria [41,42,43]. The main components of LVEO are citral (31.79%), neral (23.75%), geraniol (22.01%), and D-limonene (10.36%), inhibiting the production of the essential enzymes and causing damage to the bacterial cell walls [44,45]. However, *Pseudomonas* and *Shewanella* have high resistance to LVEO due to the presence of lipopolysaccharide in their outer membrane, which led to LVEO failing to penetrate the outer membrane. Hence, the two bacteria were the predominant bacteria in all samples. Similar results were also reported by Huang et al. [46], who found that *Pseudomonas* and *Shewanella* were the dominant spoilage bacteria identified in grass carp (*Ctenopharyngodon idellus*) fillets during chilled storage treated with oregano essential oil. Other key microbial genera, such as *Acinetobacter,* increased for CK and LYC-0.60%LVEO samples from day 0 to the 9th day and then decreased. However, the richness of *Acinetobacter* decreased for LYC-0.15%LVEO and LYC-0.30%LVEO samples from day 0 to the 9th day and kept at a low level to the end. Other species like *Psychrobacter* were found at a high level at the beginning and reduced to be negligible on the 9th and 18th days.

#### 3.1.3. Changes of Bacterial Diversity in Large Yellow Croaker Samples during Refrigerated Storage

**Figure 4:**
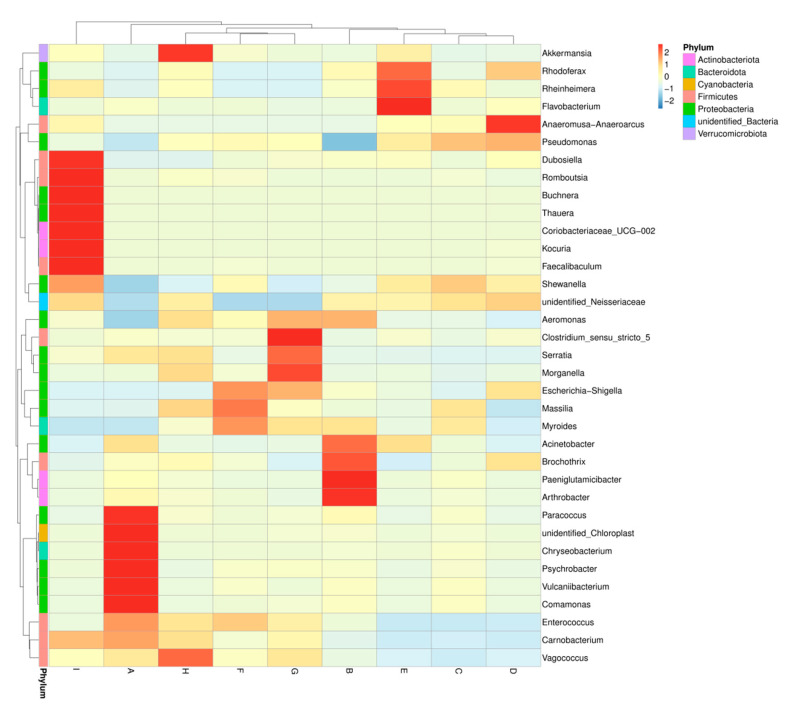
Heatmap of the changes in the microbial communities of large yellow croaker samples during refrigerated storage at 4 °C. Select the bacteria whose relative abundances were in the top 35 at the genus level obtained in this study. A: fresh; B: CK on the 9th day; C: LYC-0.15%LVEO on the 9th day; D: LYC-0.30%LVEO on the 9th day; E: LYC-0.60%LVEO on the 9th day; F: CK on the 18th day; G: LYC-0.15%LVEO on the 18th day; H: LYC-0.30%LVEO on the 18th day; I: LYC-0.60%LVEO on the 18th day.

A heatmap on the relative abundance of bacteria under the top 35 genus-level phylotypes was constructed to directly reflect the differences in bacterial composition and dynamics of large yellow croaker samples during refrigerated storage (Figure 4). The dominant bacterial genera differed among treatments. The most abundant genera of bacterial community in fresh samples were *Parococcus*, *Chryseobacterium*, *Psychrobacter*, *Vulcaniibacterium,* and *Comamonas*. However, it turned into more complex distribution in samples during refrigerated storage. Regarding the bacterial community dynamics, the five most abundant genera for CK were *Arthrobacter*, *Paeniglutamicibacter*, *Brochothrix*, *Acinetobacter* and *Aeromonas*. It switched into *Rhodoferax*, *Rheinheimera*, *Flavobacterium*, *Anaeromusa-Anaeroarcus*, and *Pseudomonas* for LVEO-treated samples. For samples stored at 4 °C on the 18th day, *Clostridium*, *Morganella,* and *Serratia* for LYC-0.15%LVEO samples; *Akkermansia* and *Vagococcus* for LYC-0.30%LVEO samples; and *Dubosiella*, *Romboutsia*, *Buchnera*, *Thauera*, *Coriobacteriaceae*, *Kocuria,* and *Faecalibaculum* for LYC-0.60%LVEO samples showed the highest abundance (Figure 3).

### 3.2. Changes in Free Carbonyl Compounds Contents

**Figure 5:**
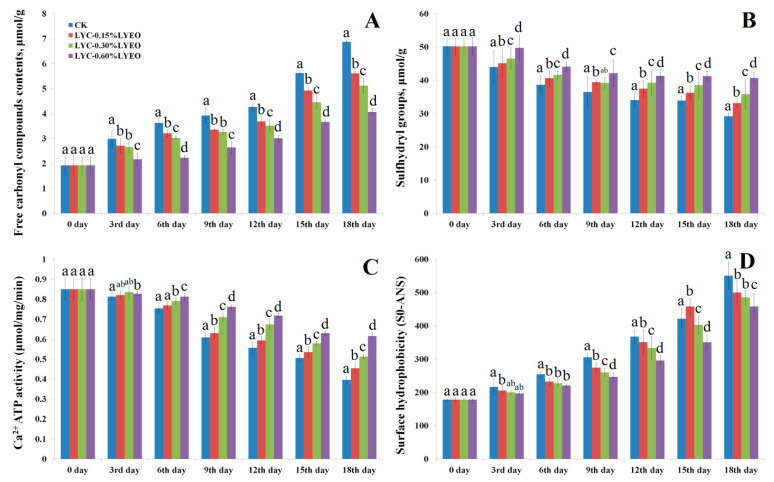
Changes in free carbonyl compounds contents (**A**), total sulfhydryl groups (**B**), Ca^2+^-ATPase activity (**C**), and surface hydrophobicity (**D**) of myofibrillar protein in large yellow croaker (*Larimichthys crocea*) during refrigerated storage. Bars represent the standard deviation (n = 3). (CK: large yellow croaker samples were packaged with FG/SA films without LVEO emulsion; LYC-0.15%LVEO: large yellow croaker samples were packaged with LBG/SA films containing 0.15% LVEO emulsion; LYC-0.30%LVEO: large yellow croaker samples were packaged with LBG/SA films containing 0.30% LVEO emulsion; and LYC-0.60%LVEO: large yellow croaker samples were packaged with LBG/SA films containing 0.60% LVEO emulsion).

Protein oxidation causes the backbone to break, forms crosslinking, and converts some amino acid residues to carbonyl compounds [47]. Therefore, the free carbonyl compounds are the main chemical products of protein oxidation. The total free carbonyl compounds contents of MPs from large yellow croaker samples increased with the prolonged storage times. The increase in total free carbonyl compounds content indicated that MPs were subjected to oxidative reactions, resulting in the oxidative degradation of proline, lysine, histidine, and arginine residues [48]. Generally, free amino acids are prone to oxidation [49]. The initial free carbonyl compounds content was 1.92 μmol/g protein, continuously increased, and reached 6.87 μmol/g protein for CK sample at the end of storage (*p* < 0.05) (Figure 5A). The LVEO treatment was found to significantly inhibit carbonyl formation in large yellow croaker samples, and their total free carbonyl compounds contents were 5.61, 5.12, and 4.06 μmol/g protein for LYC-0.15%LVEO, LYC-0.30%LVEO, and LYC-0.60%LVEO-treated samples on the 18th day, respectively. This might be associated with the lower microbial load with higher LVEO addition. Microbial proteases might cause the proteolysis, leading to the generation of peptides and the attachment of secondary lipid oxidation products, such as malondialdehyde or 4-hexyl-2 nonenal, to peptides generated [50]. Thus, the use of antioxidants is an effective strategy to retard carbonyl groups formation during protein oxidation. Farvin et al. [51] reported that the application of potato peel extract as a natural antioxidant in minced horse mackerel (*Trachurus trachurus*) led to a slower increase in carbonyl contents during chilled storage.

### 3.3. Changes in Total Sulfhydryl Contents

Sulfhydryl groups are sensitive to active hydroxyl radicals and are generally considered to be a good indicator for analyzing the degree of protein denaturation [52]. The total sulfhydryl contents of all large yellow croaker samples were significantly (*p* < 0.05) decreased during refrigerated storage (Figure 5B). LVEO acted as a novel protective agent on the total sulfhydryl contents of the MPs during refrigerated storage. The sulfhydryl contents of CK, LYC-0.15%LVEO, LYC-0.30%LVEO and LYC-0.60%LVEO treated samples decreased by 41.90%, 34.07%, 28.71%, and 19.03% on the 18th day, respectively, compared with the initial value. The decreased sulfhydryl contents may be due to the denaturation and aggregation of muscle proteins caused by cysteine sulfhydryl oxidation or disulphide interchange reactions, resulting in the formation of disulphide bonds [53]. The results showed that the decrease of the total sulfhydryl contents of LYC-0.30%LVEO and LYC-0.60%LVEO-treated samples were clearly slower than that of the CK (*p* < 0.05) during refrigerated storage, which could probably be related to the antioxidant activity of LVEO. Previous studies attributed the protective effect of different antioxidant agents on sulfhydryl groups to oxidation reactions in MPs [47,54]. The decrease in sulfhydryl contents with the concomitant formation of a disulfide bond was somewhat coincidental with the activity of Ca^2+^-ATPase. The degree of sulfhydryl oxidation is similar to that of the decrease of Ca^2+^-ATPase activity, indicating that the sulfhydryl groups played a crucial role in the activity of ATPase and the sulfhydryl oxidation led to the decrease of Ca^2+^-ATPase activity [55].

### 3.4. Changes in Ca^2+^-ATPase Activity

Even though the sulfhydryl content indicates the protein unfolding, Ca^2+^-ATPase helps to examine the protein integrity. A decrease in Ca^2+^-ATPase activity usually indicates the destruction of myosin S-1 structure [56]. Ca^2+^-ATPase activity of MPs in large yellow croaker samples exhibited a significant decrease (*p* < 0.05) during refrigerated storage (Figure 5C), which could be due to the conformational changes of myosin globular heads, protein-protein interaction, and increased ionic strength [57]. The Ca^2+^-ATPase activity of CK sample decreased with the highest speed during the refrigerated storage, indicating the degeneration of MPs. Ca^2+^-ATPase activity in fresh large yellow croaker samples was about 0.85 μmol/mg protein·min^−1^ (Figure 5C); the values decreased to 0.40, 0.45, 0.51, and 0.61 μmol/mg protein·min^−1^ for the CK, LYC-0.15%LVEO, LYC-0.30%LVEO, and LYC-0.60%LVEO-treated samples on the 18th day, respectively. It can be concluded that LYC-LVEO treatments can maintain the activity of Ca^2+^-ATPase to some extent by protecting MPs integrity. Ca^2+^-ATPase activity is closely related to the sulfhydryl groups on the myosin globular head [57]. The sulfhydryl group of cysteine is the center of the Ca^2+^-ATPase activity; therefore, the oxidation of the sulfhydryl groups on the myosin globular head is the reason for the decrease of Ca^2+^-ATPase activity [58,59]. The oxidation of sulfhydryl groups on the active site led to a decrease in Ca^2+^-ATPase activity, which had a similar trend between the sulfhydryl content and Ca^2+^-ATPase activity. In the present study, LYC-LVEO treatments could retard the oxidation of sulfhydryl groups and decrease Ca^2+^-ATPase activity in large yellow croaker samples during refrigerated storage.

### 3.5. Changes in Surface Hydrophobicity

Changes in surface hydrophobicity can be utilized to monitor the conformational changes in protein structure and be a sensitive indicator of subtle changes in physical and chemical states of MPs [60]. The surface hydrophobicity of fresh sample was approximately 177.96 and increased significantly (*p* < 0.05) to 550.61 in the CK samples on the 18th day, which is significantly higher (*p* < 0.05) than 499.90, 485.20, and 458.13 in LYC-0.15%LVEO, LYC-0.30%LVEO, and LYC-0.60%LVEO-treated samples on the 18th day, respectively (Figure 5D). However, the results between the three treatments (LYC-0.15%LVEO, LYC-0.30%LVEO, and LYC-0.60%LVEO) had no significant difference (*p* > 0.05). The results demonstrated that the LYC-LVEO treatments effectively retarded the increase of surface hydrophobicity in the large yellow croaker samples during refrigerated storage. Lower surface hydrophobicity demonstrated that less hydrophobic protein was bound to fluorescent probes, reflecting less exposure of hydrophobic groups and less protein denaturation [60]. The increase in surface hydrophobicity of MPs during refrigerated storage can be ascribed to the proteins unfolding and the exposure of hydrophobic aliphatic and aromatic amino acids [61].

### 3.6. Secondary Structure Changes Analysed by Raman Spectroscopy

Raman spectroscopy can reflect the secondary structure of MPs [62], and the contents of different types of secondary structure are usually determined by analyzing the amide I band. In order to explore the secondary structure changes of MPs in large yellow croaker samples during refrigerated storage, this measurement was performed and the spectra contained four secondary structures, α-helices (1645–1657 cm^−1^), β-sheets (1665–1680 cm^−1^), β-turns (near 1680 cm^−1^), and random coils (1660–1665 cm^−1^) [63]. In denaturation of MPs, the α-helices shifted to β-sheets and β-turns, and then β-sheets shifted to random coil, which were ascribed to the breakdown of hydrogen bonds and the increase of hydrophobicity [38]. Therefore, α-helices were a kind of regular structure, with a higher content of α-helices indicating more stability in secondary structure. The proportion of α-helices in the CK samples significantly decreased to 63.46% on the 18th day (Table 2), while the proportion of random coils increased dramatically to 58.64%, compared with fresh samples (*p* < 0.05). However, the ratio of α-helices in LVEO-treated samples decreased by 33.25–45.29% on the 18th day. The changing trend of secondary structure in the CK samples was similar to that of the LVEO-treated samples, indicating that the α-helices transformed into random coils. The transition from α-helices to random coils is mainly for the increase of hydrophobicity and the destruction of the hydrogen bonds, which keep the stability of α-helices structure, leading to the disintegration of the α-helix structure [64]. The decreased α-helices contents and the increased random coils contents usually result from the denaturation and unwrapping of protein molecules [65]. The α-helix contents in LVEO-treated samples were higher than that in CK samples at each sampling time. The α-helices, β-turns, and random coils contents in the three LVEO treated samples had no significant differences in the three LVEO-treated samples (*p* > 0.05) during refrigerated storage. There were less α-helices transforming into random coils in LVEO-treated samples, indicating that the LVEO-treated samples had more stable MPs structure. Hydrogen bond is the main interaction force maintaining the stability of the α-helical structure. The decrease of α-helices content in the MPs was related to the gradual breaking of hydrogen bonds and the partial unfolding of the helical structure [66,67]. In the present research, the decrease in α-helices with hydrogen bond fracture was induced to expose the hydrophobic groups, resulting in the increase in hydrophobic interactions. The antioxidant compounds in LVEO exhibited a protective effect against lipid oxidation, inhibiting the formation of secondary lipid oxidation products and protein oxidation and maintaining the protein conformation. Furthermore, polyphenols in LVEO interacting with protein may also lead to structure stability (higher α-helical content and lower random coils content) than the CK samples through hydrophobic interactions and hydrogen bonding [68].

### 3.7. Tertiary Structure Changes Analysed by IFI

**Figure 6:**
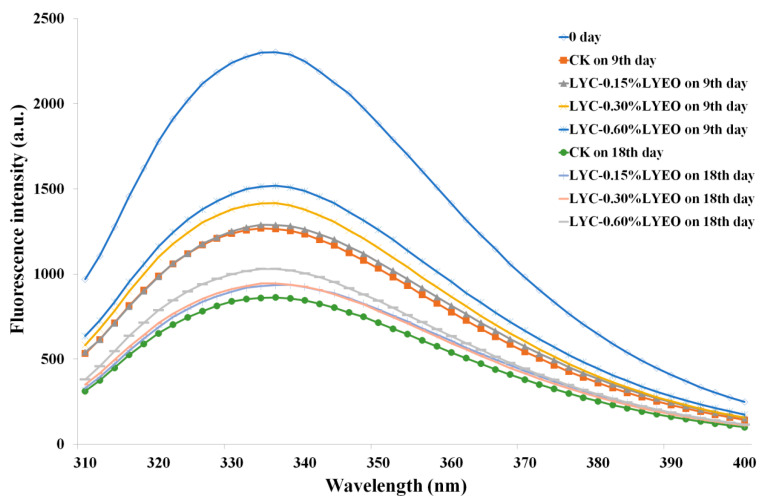
Changes in intrinsic fluorescence intensity (IFI) of myofibrillar protein in large yellow croaker (*Larimichthys crocea*) during refrigerated storage. (CK: large yellow croaker samples were packaged with FG/SA films without LVEO emulsion; LYC-0.15%LVEO: large yellow croaker samples were packaged with LBG/SA films containing 0.15% LVEO emulsion; LYC-0.30%LVEO: large yellow croaker samples were packaged with LBG/SA films containing 0.30% LVEO emulsion; and LYC-0.60%LVEO: large yellow croaker samples were packaged with LBG/SA films containing 0.60% LVEO emulsion).

IFI is a well-established technique for tracking tertiary structure changes in MPs during refrigerated storage. The IFI of MPs mainly contributed to by tryptophan (Trp) residues is sensitive to the micro-environments [69]. When Trp and other hydrophobic amino acid residues are embedded in the protein core, the fluorescence intensity is higher [54]. The maximum IFI of MPs was found at 330–340 nm for each sample in the present research (Figure 6). Storage time resulted in a shift of *λ*_max_ from 336.3 nm (on 0 day) to 333.7, 334.0, 336.3, and 336.3 nm for CK, LYC-0.15%LVEO, LYC-0.30%LVEO, and LYC-0.60%LVEO samples on the 9th day, respectively. This result indicated that the fluorophores were exposed to the hydrophilic environment. While the *λ*_max_ of CK, LYC-0.15%LVEO, LYC-0.30%LVEO, and LYC-0.60%LVEO samples on the 18th day showed a slight shift to 336.0, 338.3, 338.7, and 339.3 nm, respectively, the result showed that some of the tryptophan residues were exposed to a polar environment, and the exposure of hydrophobic groups and the hydrophobic interaction led to the formation of protein aggregates [70]. The unfolding and aggregation of MP in large yellow croaker samples that occurred during refrigerated storage may destroy hydrophobic interactions. The IFI of Trp in the large yellow croaker samples decreased significantly compared with the CK sample during refrigerated storage. The IFI decreased with the increase storage time. The trend indicated that refrigerated storage enhanced the polar environment around the Trp residues, which may produce a shielding effect to reduce the overall fluorescence [71]. At the end of storage, the IFI in CK, LYC-0.15%LVEO, LYC-0.30%LVEO, and LYC-0.60%LVEO samples decreased by 62.54%, 59.35%, 59.33%, and 55.28%, respectively, compared to the initial value. The IFI of large yellow croaker treated with LYC-LVEO bioactive coatings was significantly higher than that in CK after 18 days (*p* < 0.05) and decreased protein degeneration and tertiary structure changes during refrigerated storage.

### 3.8. Morphological Changes Detected by TEM

**Figure 7:**
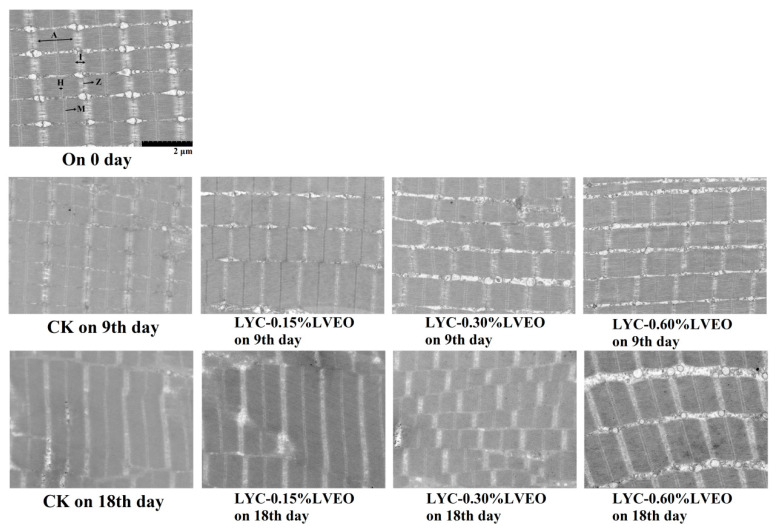
Transmission electron microscopy (TEM) micrographs of large yellow croaker (*Larimichthys crocea*) during refrigerated storage. (CK: large yellow croaker samples were packaged with FG/SA films without LVEO emulsion; LYC-0.15%LVEO: large yellow croaker samples were packaged with LBG/SA films containing 0.15% LVEO emulsion; LYC-0.30%LVEO: large yellow croaker samples were packaged with LBG/SA films containing 0.30% LVEO emulsion; and LYC-0.60%LVEO: large yellow croaker samples were packaged with LBG/SA films containing 0.60% LVEO emulsion).

The ultrastructure of the muscle determines the structure of the fish and reflects the changes in MPs. Therefore, a deep investigation on muscle ultrastructure played an important role in exploring the potential effect of LYC-LVEO treatments on the degradation of MPs. As shown in Figure 7, the fresh samples had clear Z- and M-lines as well as H-, A-, and I-bands, and the refrigerated storage changed the sarcomere structure of the samples. At the end of storage, the Z- and M-lines of CK samples were fuzzy and they were not able to identify the sarcomere structure in some cases to the full extent. The boundaries of H-, A-, and I-bands became blurred, and the sarcomere integrity was damaged, which was related to the degradation of titin, playing an important role in the stability of thick filaments and the connection between sarcomere [34]. Sarcomere is the basic unit of contraction of striated muscles, which are stacked in the muscle tissue. The sarcomere contains many actin (thin) and myosin (thick) filaments assembling into parallel bundles. Myosin and actin filaments are maintained within the sarcomere by the M and Z lines, respectively. The sarcomere extends from one Z-line to the next, with the M-line in the center of the H-zone [72]. Yang et al. [34] suggested that the degradation of desmin and titin could cause the destruction of Z-disk. In addition, the severe degradation inevitably led to the disintegration of A- and I-bands due to the connection between M-line and Z-disk. The sarcomere structure of CK samples appeared completely destroyed. Previous research showed that the reduction of the mechanical constraints of the fiber nets could result in the conversion of secondary structure by reducing the hydrogen bond’s force [73]. The disordered arrangement of MPs was consistent with the results of Raman spectroscopy parameters. However, the ultrastructures in the LVEO-treated samples were still regular in appearance. The light I-bands and dark A-bands were distinct in the large yellow croaker samples from LYC-0.30%LVEO and LYC-0.60%LVEO samples, and Z-disk was also in good shape.

## 4. Conclusions

*Pseudomonas* and *Shewanella* were the two major genera identified during refrigerated storage according to the high throughput sequencing analysis results. The obviously decreased degradation of MPs during refrigerated storage was observed in the presence of LVEO treatments. Compared with the CK sample, LVEO-active coatings exerted significant protective effects against MPs oxidation, including the restriction of free carbonyl compounds formation and surface hydrophobicity, and a smaller reduction in the total sulfhydryl groups and Ca^2+^-ATPase activity. The secondary protein structure was effectively protected by LVEO coatings with the increased α-helices and decreased random coils contents. IFI measurements revealed a slighter decrement of the conformational changes of MPs. What is more, the treated MPs ultrastructure of large yellow croaker was still in good shape with better light I-bands, dark A-bands, and Z-disk at the end of storage. In summary, the antibacterial effect of LVEO-active coatings played an important role in the changes of bacterial diversity and MPs.

## Figures and Tables

**Table 1:** Biodiversity analysis of 16S rRNA sequences in bacterial V4 region in large yellow croaker (*Larimichthys crocea*) samples during refrigerated storage at 4 °C.

Sample Treatments	OTUs ^1^	Shannon ^2^	Simpson	Chao1 ^3^	ACE ^4^	Good’s Coverage ^5^
A ^6^	322	4.811	0.888	425.722	459.919	0.990
B	247	4.045	0.874	374.918	398.132	0.990
C	265	3.568	0.795	421.004	442.147	0.989
D	234	3.180	0.733	373.916	391.066	0.990
E	258	3.715	0.799	386.996	410.779	0.990
F	253	3.805	0.819	367.052	403.958	0.990
G	141	3.681	0.809	178.549	186.535	0.996
H	195	3.987	0.834	281.772	293.864	0.993
I	251	3.822	0.832	395.197	408.786	0.990

^1^ Operational taxonomic units (OTUs) were the classified operational units. ^2^ Shannon defining as the diversity index. ^3^ The diversity indexes included Chao representing the species richness. ^4^ The abundance-based coverage (ACE) indicating the uniformity. ^5^ Good’s coverage was calculated using QIIME software at a level of similarity of 97%. ^6^ A: fresh; B: CK on the 9th day; C: LYC-0.15%LVEO on the 9th day; D: LYC-0.30%LVEO on the 9th day; E: LYC-0.60%LVEO on the 9th day; F: CK on the 18th day; G: LYC-0.15%LVEO on the 18th day; H: LYC-0.30%LVEO on the 18th day; I: LYC-0.60%LVEO on the 18th day.

**Table 2:** Changes in the secondary structure contents of myofibrillar protein in large yellow croaker (*Larimichthys crocea*) during refrigerated storage.

Samples	α-Helices	β-Sheets	β-Turns	Random Coils
Fresh	20.8%	12.5%	28.5%	38.2%
CK on 9th day ^1^	27.6%	13.3%	20.4%	38.7%
LYC-0.15%LVEO on 9th day	8.4%	15.5%	20.3%	55.8%
LYC-0.30%LVEO on 9th day	21.0%	12.0%	26.6%	40.4%
LYC-0.60%LVEO on 9th day	17.0%	20.6%	33.0%	29.4%
CK on 18th day	7.6%	10.1%	21.7%	60.6%
LYC-0.15%LVEO on 18th day	8.3%	15.6%	20.6%	55.5%
LYC-0.30%LVEO on 18th day	10.2%	15.4%	23.5%	50.9%
LYC-0.60%LVEO on 18th day	10.9%	15.6%	22.3%	51.2%

^1^ CK: large yellow croaker samples were packaged with FG/SA films without LVEO emulsion; LYC-0.15%LVEO: large yellow croaker samples were packaged with LBG/SA films containing 0.15% LVEO emulsion; LYC-0.30%LVEO: large yellow croaker samples were packaged with LBG/SA films containing 0.30% LVEO emulsion; and LYC-0.60%LVEO: large yellow croaker samples were packaged with LBG/SA films containing 0.60% LVEO emulsion.

## Data Availability

Data are contained within the article.
